# Comparison of a hand-held high-end resolution infrared thermography (FLIR P640) and a smartphone infrared thermographic device (FLIR One) for the assessment of skin surface temperature after anaesthetising the median nerve in Healthy horses

**DOI:** 10.1371/journal.pone.0309603

**Published:** 2024-08-30

**Authors:** Gustavo Ferlini Agne, Kellie Adamson, Leah McGlinchey, Olena Kravchuk, Luiz Santos, John Schumacher

**Affiliations:** 1 School of Animal and Veterinary Science, The University of Adelaide, Roseworthy, South Australia; 2 Department of Clinical Sciences, Auburn University College of Veterinary Medicine, Auburn, AL, United States of America; 3 School of Agriculture, Food and Wine, The University of Adelaide, Adelaide, South Australia; 4 School of Biodiversity, One Health & Veterinary Medicine, The University of Glasgow, Glasgow, Bearsden, United Kingdom; Cairo University Faculty of Veterinary Medicine, EGYPT

## Abstract

Accuracy of a median nerve block is normally assessed by testing skin sensitivity on the medial and dorsal aspects of fetlock and pastern. The present study evaluated subjective and objective analysis of skin surface temperature obtained with two different infrared (IR) thermography cameras (a high-end [FLIR P640] and a smartphone IR thermography device [FLIR One®]) before and after anaesthesia of the median nerve. Thermographic images were obtained at 0, 30, 60 and 90 minutes after performing a median nerve block with 2% mepivacaine hydrochloride. The subjective analysis of thermographic images using the FLIR P640 camera found assessors had >50% agreement for the presence of a nerve block (p<0.01) based on assessment of skin temperature within the expected dermatome of the median nerve. The objective analysis found skin temperature increases in the treated leg using the FLIR One® in the dorsal fetlock, dorsal pastern and medial pastern at 60 minutes, and the lateral pastern at 90 minutes (p<0.05). The treated leg, imaged using the FLIR P640 camera, had increases in skin temperature at the medial aspect of the fetlock at 60 minutes and lateral pastern at 90 minutes (p<0.05). Images obtained with the P640 camera had higher resolution and finer thermal detail. The images obtained with the FLIR One® camera had a wider temperature range with overall higher temperature measurements than the images obtained using the P640 camera (p<0.001). Skin temperatures in horses should be interpreted with caution when using the FLIR One® camera. Furthermore, the FLIR One® device detected an increase in skin surface temperature in both treated and non-treated legs and should not be used for assessment of a median nerve block. Infrared thermography appears to be useful for determining the presence of a high regional nerve block such as the median nerve block by observing increased temperatures of the skin surface after perineural anaesthesia. Further studies with a larger sample size as well as investigating the use of thermography for assessment of other regional nerve blocks are warranted.

## 1. Introduction

It is often difficult to judge the accuracy of a high regional nerve block (i.e., anaesthesia of the median, ulnar, peroneal, or tibial nerve) in the horse. After a high regional nerve block, skin can be tested for loss of sensation at a specific site on the limb for each nerve; unfortunately, this method of testing may yield misleading information for several reasons: [1] the horse may be stoic and show little reaction to noxious stimulation of skin, [2] the region of skin desensitized may vary somewhat among horses [1], and [3] some horses react violently to the slightest provocation, making a positive reaction to skin testing difficult to interpret. Also, after performing a regional nerve block, presence, or absence of skin sensitivity within the dermatome of the nerve may not correlate with presence or absence of deep pain [2]. A positive response to a nerve block (i.e., resolution of lameness) is good evidence that the nerve was anaesthetised, but a negative response may mean that the source of pain causing lameness was not in the region supplied by that nerve or that anaesthesia of the nerve was not achieved; the reliability of diagnostic analgesia depends upon the ability to make this distinction. A simple, reliable method for determining the accuracy of a high regional nerve block would make the use of high regional nerve blocks a more attractive and useful procedure for lameness examination.

The nerves of the skin can be divided into two categories: sensory and autonomic. The sensory nerves are for transmission of the sensation of temperature, pain, itch, light, touch, pressure, and proprioception; whereas the autonomic nervous system controls the tone of cutaneous blood vessels and skin glands. Given that local anaesthetic is deposited around sensory and autonomic nerves, both nerve categories are simultaneously anaesthetised resulting in vasodilation with an increase in blood flow to the skin and a decrease in the skin sensation to touch and pain [3]. Vasodilation of dermal blood vessels may cause a temperature increase within the dermatome of the anesthetized nerve. Infrared thermographic imaging of the skin has been used successfully to detect the accuracy of high regional nerve blocks in people. A study evaluating the thermographic changes of the skin after a sub gluteal sciatic nerve block in people, reported a significant increase in temperature of the toes and foot [4]. In the same study, a positive correlation was observed between pinprick testing and skin temperature measurements. Temperature increase in portions of the lower leg after a median nerve block in horses has been described [3,5], however no studies have been performed to objectively confirm this.

A variety of cameras are available for thermography evaluation including high-end resolution handheld devices and recently, smartphone infrared (IR) thermography devices. Because a high-end resolution IR thermography camera can be cost prohibitive, these devices might not be a viable option for many veterinary practitioners. A more affordable thermographic device such as the smartphone IR thermography device (FLIR One®) could be an alternative device for evaluating skin temperature if it can provide information similar to that obtained with a high-end resolution camera. Comparison of the usefulness of thermographic images obtained with high-end resolution cameras to images obtained using smartphone IR thermography devices in horses is currently lacking. The objectives of this study were to determine [1], if thermography can be used to determine the success of a median nerve block both objectively and subjectively, and [2], if the FLIR One® iPhone attachment can be used as an alternative to an expensive, high-end resolution camera for IR thermography for detecting substantial changes in skin temperature after anaesthesia of the median nerve in the horse.

## 2. Material and methods

### 2.1. Horses

A total of six healthy adult horses from the Auburn University teaching herd were enrolled in the study. Horses age ranged from 10 to 24 years with a median age of 16.5 years and weight ranged from 450 to 540kg with a median weight of 521kg. Horses were considered healthy based on physical examination (rectal temperature less than 38.5 degrees Celsius, pink and moist mucous membranes, capillary and jugular refill time of less than 2 seconds, normal cardiothoracic and gastrointestinal auscultation, no evidence of ocular or nasal discharge and no swelling of lymph nodes) performed prior to enrolment into the study which was approved by the University’s Institutional Animal Care and Use Committee (IACUC #2017–3009).

### 2.2. Median nerve block

Ten millilitres (10 mL) of 2% mepivacaine hydrochloride was injected at the junction of the caudomedial border of the radius and the ventral edge of the superficial pectoral muscle using a 20-gauge, 1-inch needle directed in a caudolateral direction as previously described [6]. All horses tolerated the procedure which was performed without sedation and with the use of nose twitch. Cutaneous sensation was tested in both forelimbs by three evaluators who were unaware of which limb was the treated limb. Skin sensitivity was evaluated before, 30, 60 and 90 minutes after performing the nerve block. Skin sensitivity was evaluated by pinching the skin on the medial aspect of the pastern of each forelimb with haemostatic forceps.

### 2.3. Infrared thermography image acquisition

**Fig 1:**
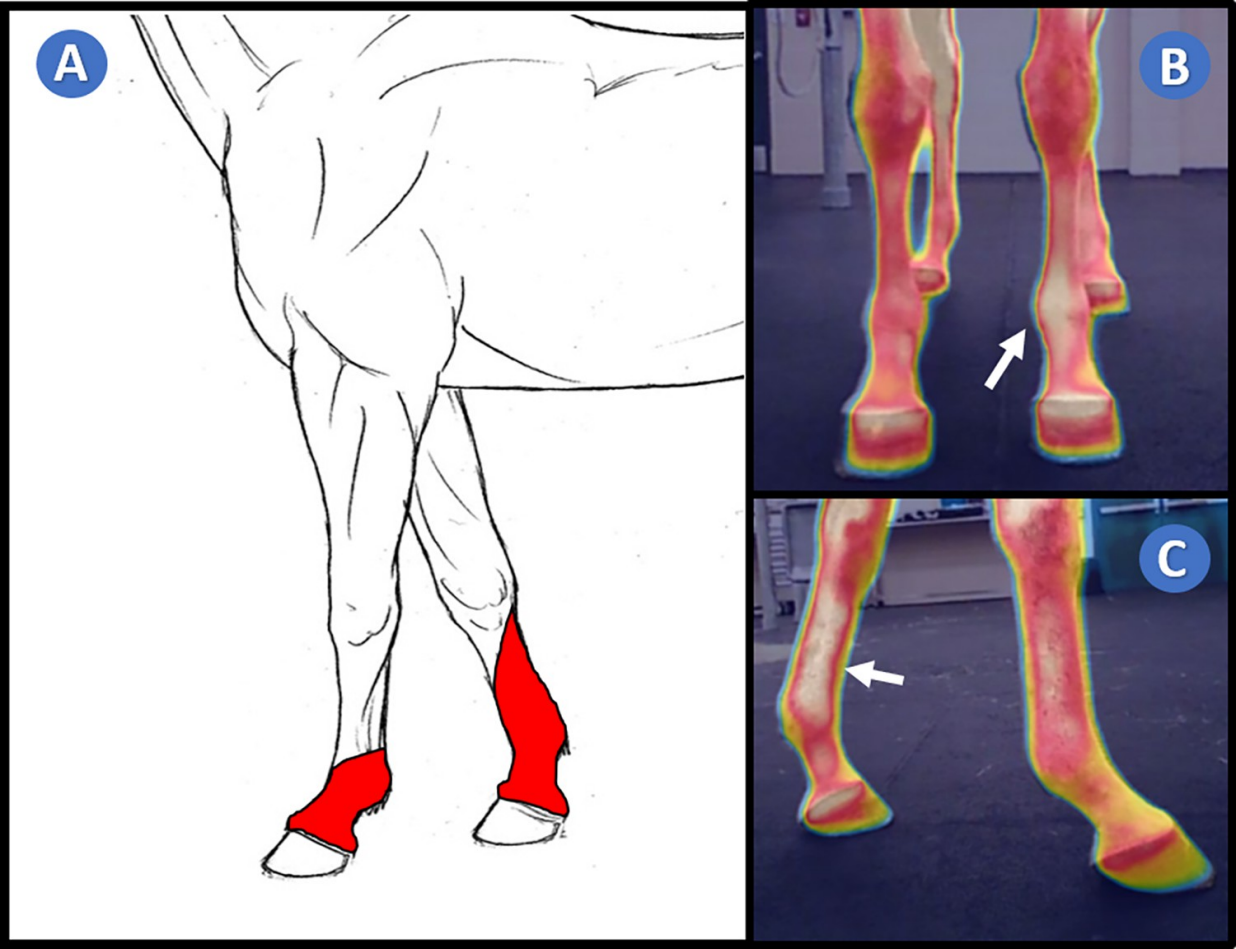
Median nerve dermatome. (A) Anatomical demarcation in red of the median nerve dermatome adapted from Budras et al. [7]; (B) Dorso-palmar merged infrared thermography and standard imaging depicting an increase in focal heat in the dorsal fetlock and pastern median nerve dermatome (arrow); (C) Latero-medial merged infrared thermography and standard imaging depicting an increase in focal heat in the medial metacarpal, fetlock and pastern median nerve dermatome (arrow).

**Fig 2:**
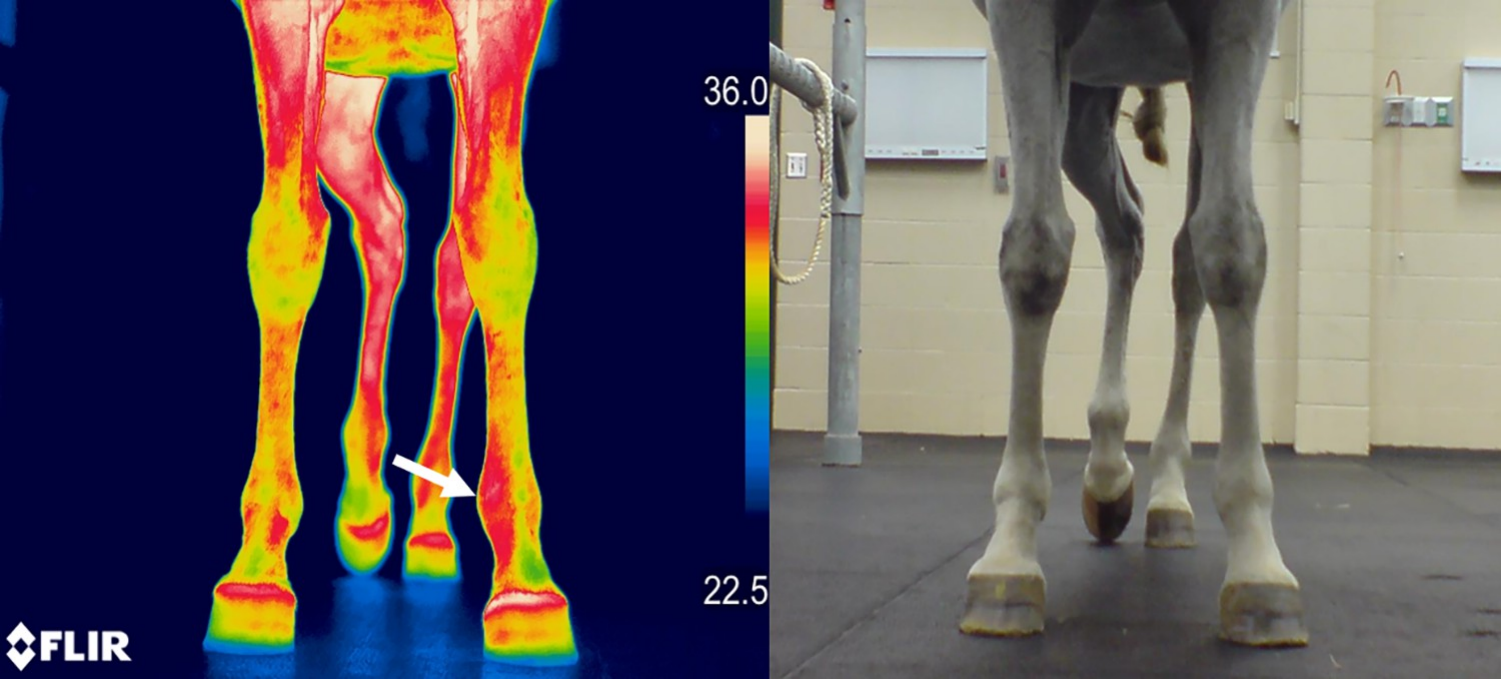
Infrared thermography of median nerve dermatome. Dorsal-palmar thermographic image and photographic image obtained with the P640 camera demonstrating an increase in skin temperature on the medial aspect of the left fetlock (arrow) within the dermatome of the median nerve of the left limb after the nerve was blocked.

The thermographic images were obtained by one of the authors (GFA). The images contained the dorso-palmar, lateromedial and mediolateral views of both forelimbs obtained immediately before the nerve block (T = 0) and 30, 60 and 90 minutes after the nerve block. Infrared thermography imaging included areas of the median nerve dermatome, i.e. the dorsal, lateral and medial aspects of fetlock and pastern (Figs 1 and 2). Images were obtained with two uncooled focal array microbolometer devices: a high-end resolution thermographic camera (FLIR P640—autofocus; accuracy: ± 2°C or 2% of the reading; image resolution: 640 × 480 pixels; frame rate: 30 Hz) and a smartphone thermographic camera device (FLIR One®—fixed focus; accuracy: ± 3°C or 5% of the reading; image resolution: 160 × 120 pixels; frame rate: 8.7 Hz) attached to an iPhone 5S®. Both cameras were turned on for warmup for at least 5 minutes prior to image acquisition. The FLIR P640 camera had a history of regular quality assurance (QA) testing against a blackbody in a research setting whilst the FLIR One camera had been recently purchased with a QA certificated from the manufacturer. The cameras were consistently positioned two metres from a marker on the ground indicating the point where the horses’ forelimbs were positioned for imaging. All the images were obtained in a climate-controlled room with constant relative humidity of 50% and temperature of 21 degrees Celsius. All horses were groomed 60 minutes prior to image acquisition and were given 20 minutes to acclimatise to the environment.

### 2.4 Subjective infrared thermography evaluation

A random selection of 18 IR thermography images obtained using the FLIR P640 camera were provided to assessors (GFA, LS, KA). The images allowed comparison between the baseline image (obtained prior to the median nerve block) and either a 30-, 60- or 90-minutes post nerve block image. Assessors were blinded to which limb was blocked and recorded whether they could visualise a subjective increase in skin temperature in the region of the dermatome of the median nerve compared to baseline by indicating “yes” or “no”, and which forelimb they believed had received the median nerve block.

### 2.5 Objective infrared thermography evaluation

**Fig 3:**
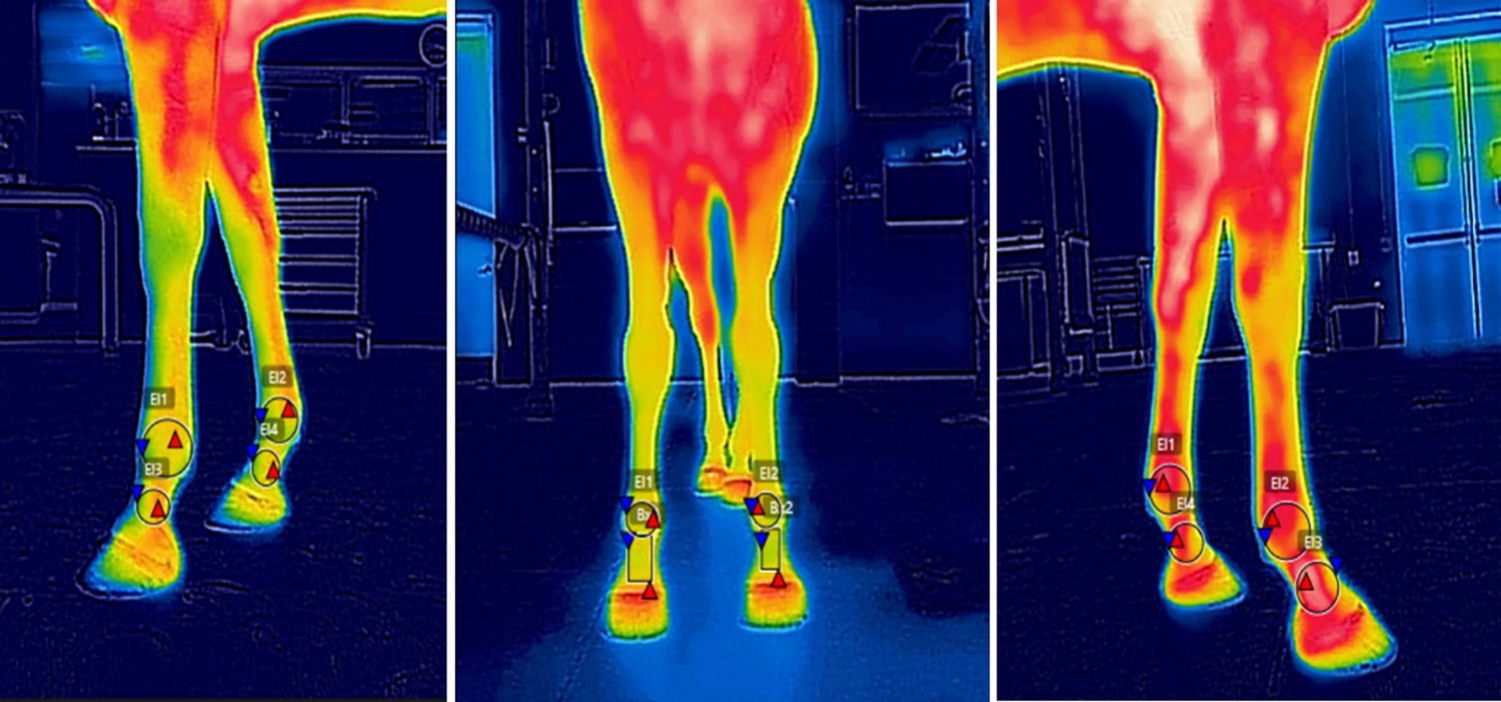
FLIR One® Regions of interest (ROI). Dorsal and lateral thermographic views with the ROI of the pastern and fetlock obtained using the FLIR One® camera.

**Fig 4:**
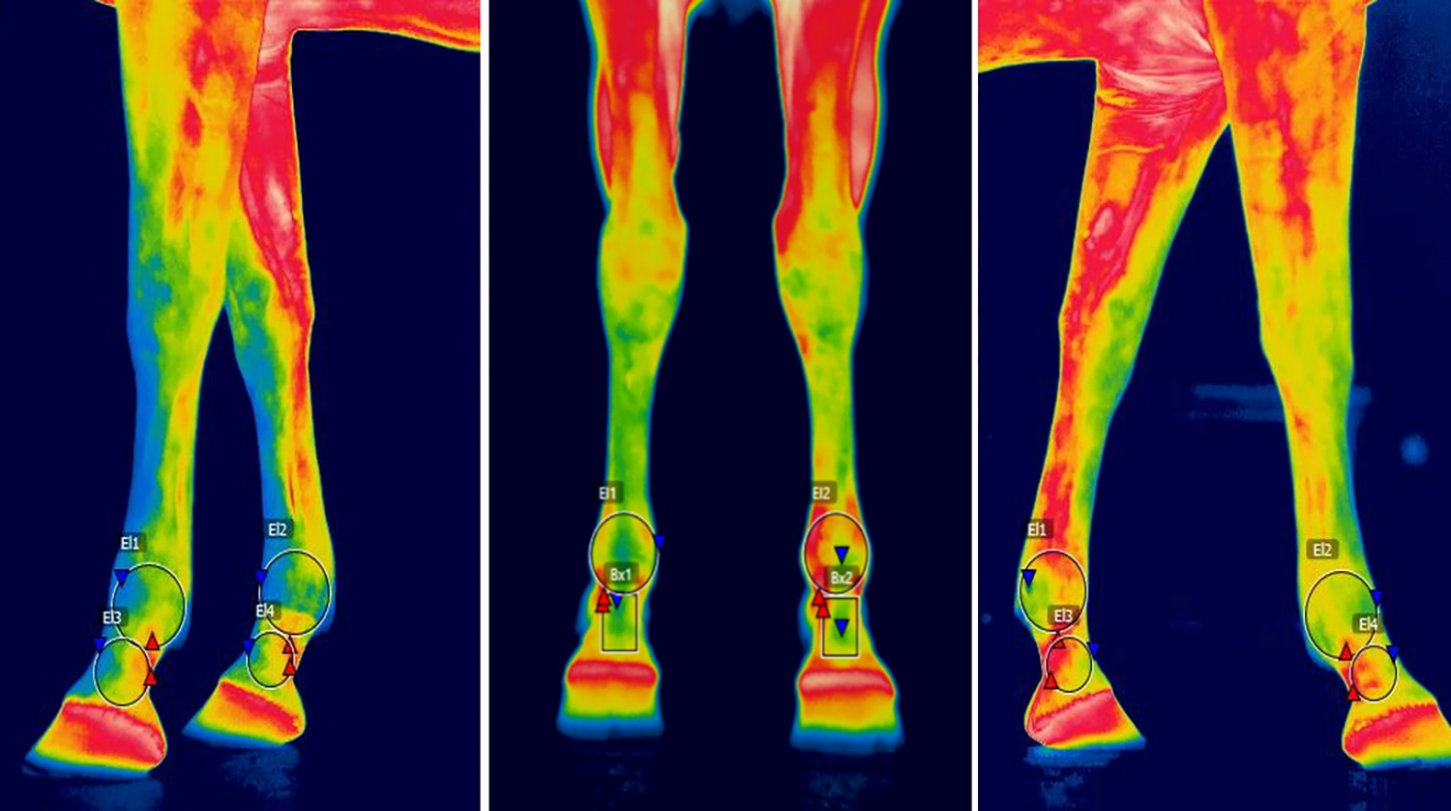
FLIR P640 Regions of interest (ROI). Dorsal and lateral thermographic views with the regions of interest of the pastern and fetlock obtained using the FLIR P640 camera.

The FLIR Tools Software (v5.2, FLIR Systems Inc.) was applied to selected areas for temperature assessment. An emissivity of 0.98 was set as the default in the FLIR software as per previous recommendations [8]. Skin surface temperature measured with the software provided the minimum, maximum, and average temperature for all the selected anatomical regions of interest (ROI) in both forelegs (Figs 3 and 4).

### 2.6 Statistical analysis

Minimum, maximum and average skin surface temperature were used for objective data analysis and normality assessed via D’Agostino & Pearson as well as Shapiro-Wilk tests. Subjective analysis for the presence of a nerve block from a random selection of images was analysed via a one sample binomial test. Cohen’s kappa agreement test was used for the assessment of the side of blockage (0.81–1.00 indicating perfect agreement, 0.61–0.80 substantial agreement, 0.41–0.60 moderate, 0.21–0.40 fair, 0.01–0.20 slight and <0 no agreement) [9]. Wilcoxon signed rank exact paired test was used to determine comparative differences in skin surface temperature between the two cameras and between non-blocked and blocked legs for each camera at each time point. Determination of statistical difference in skin temperature between baseline (prior to nerve block) and the different time points after the nerve block was determined with the use of Friedman repeated measures analysis. Values with statistical significance were then compared with Dunn’s pairwise post-hoc analysis. Correlation between the IR smartphone device and the high-end resolution IR camera were compared using Spearman’s correlation test with a value of 1 suggesting perfect correlation and 0 suggesting no correlation [10]. Statistical significance was set with p-values of <0.05 for Wilcoxon tests, Friedman tests and Spearman’s correlation and <0.01 for the subjective analysis results. The above analyses were performed using the statistical software package IBM SPSS Statistics for Windows, Version 20.0 (IBM Corp, New York, USA) and GraphPad Prism 9 (GraphPad Prism 6, GraphPad Software Inc, CA, USA)

## 3. Results

### 3.1. Subjective evaluation

Based on skin sensitivity testing, the three evaluators believed that the median nerve was successfully anaesthetised in all horses. When the three evaluators subjectively examined the thermographic images for evidence of a successful nerve block, assessor 1 had 100% agreement with skin sensitivity testing; assessor 2 had 94.4% agreement and assessor 3 had 83.3% agreement (Table 1). Based on subjective evaluation of thermographic images the three assessors had >50% agreement for the presence of a nerve block (p<0.01). When determining which limb had received the nerve block, assessor 1 had near perfect agreement with kappa coefficient of 0.88. Assessor 2 had moderate agreement with kappa coefficient of 0.532 and assessor 3 had a lack of agreement with a kappa coefficient of 0.299. Combined, all three assessors had a significant agreement with a kappa coefficient of 0.52 in regard to which leg had received the nerve block (Table 2).

**Table 1:** One sample binomial test for successful detection of a median nerve blockage by subjective analysis of 18 randomly selected infrared thermography images performed by three different assessors.

Assessor	Sample Size	Successes	Proportion	p-Value
1	18	18	1	
2	18	17	0.944	<0.001*
3	18	15	0.833	0.008*

(* = significance p<0.01).

**Table 2:** Cohen’s kappa agreement test for successful detection for the side of the median nerve block by subjective analysis of 18 randomly selected infrared thermography images performed by three different assessors.

Assessor	Actual	Expected	Kappa Coefficient	p-Value
1	0.944	0.539	0.880	<0.001*
2	0.778	0.525	0.532	0.016
3	0.667	0.525	0.299	0.11
All 3	0.741	0.459	0.521	<0.001*

Kappa Coefficient: 0.81–1.00 indicating perfect agreement, 0.61–0.80 substantial agreement, 0.41–0.60 moderate, 0.21–0.40 fair, 0.01–0.20 slight and <0 no agreement.

(* = significance p<0.01).

### 3.2. Objective skin temperature evaluation

**Fig 5:**
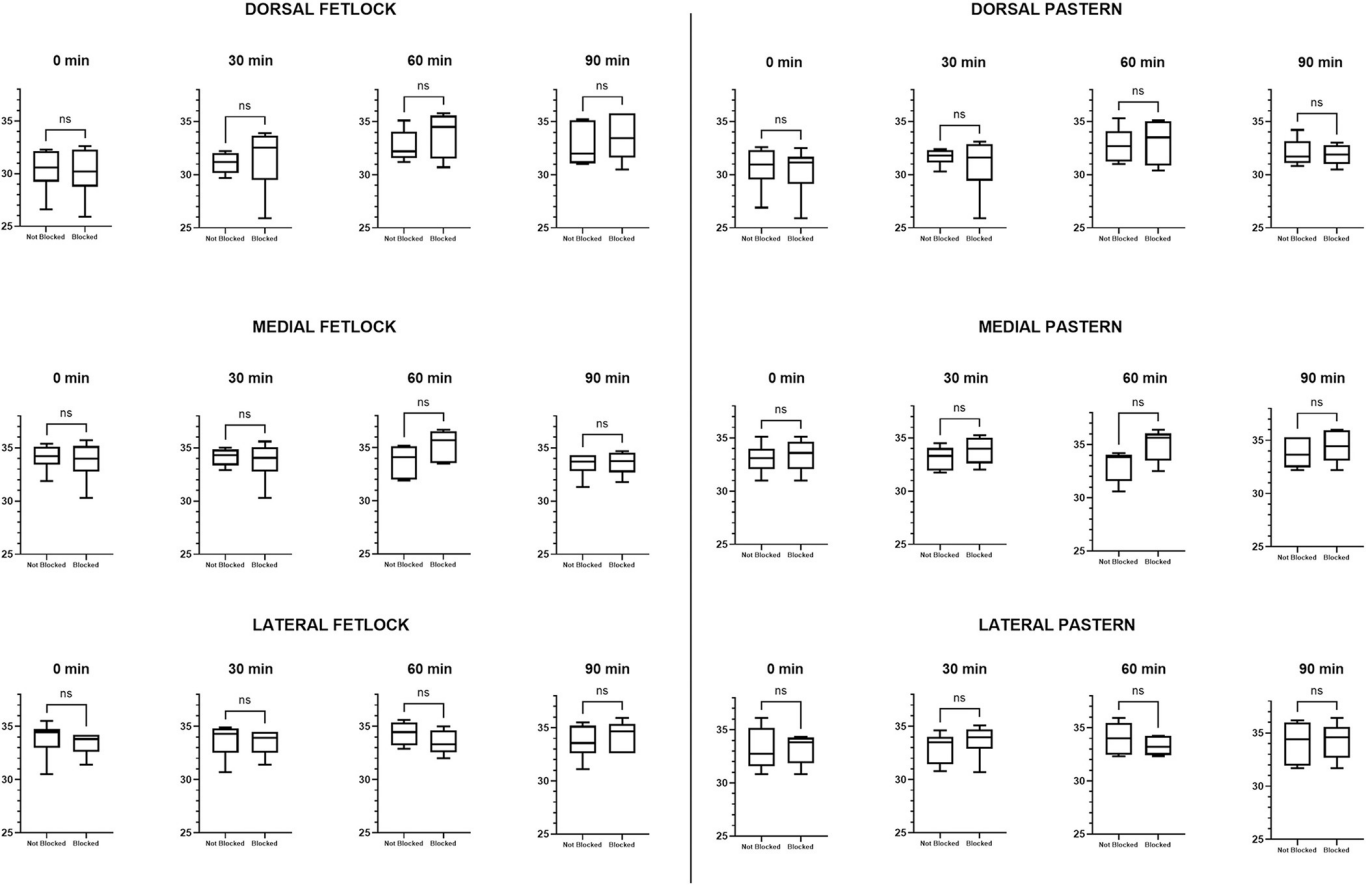
FLIR One® maximum skin temperature between limbs. Box and whisker plots showing the maximum skin temperature in each limb for the areas of the dermatome of the median nerve taken by the FLIR One® camera. The line indicates the median, the box the interquartile range of 25–75% and the whiskers the minimum and maximum. (ns = no significance, * = significance p<0.05).

**Fig 6:**
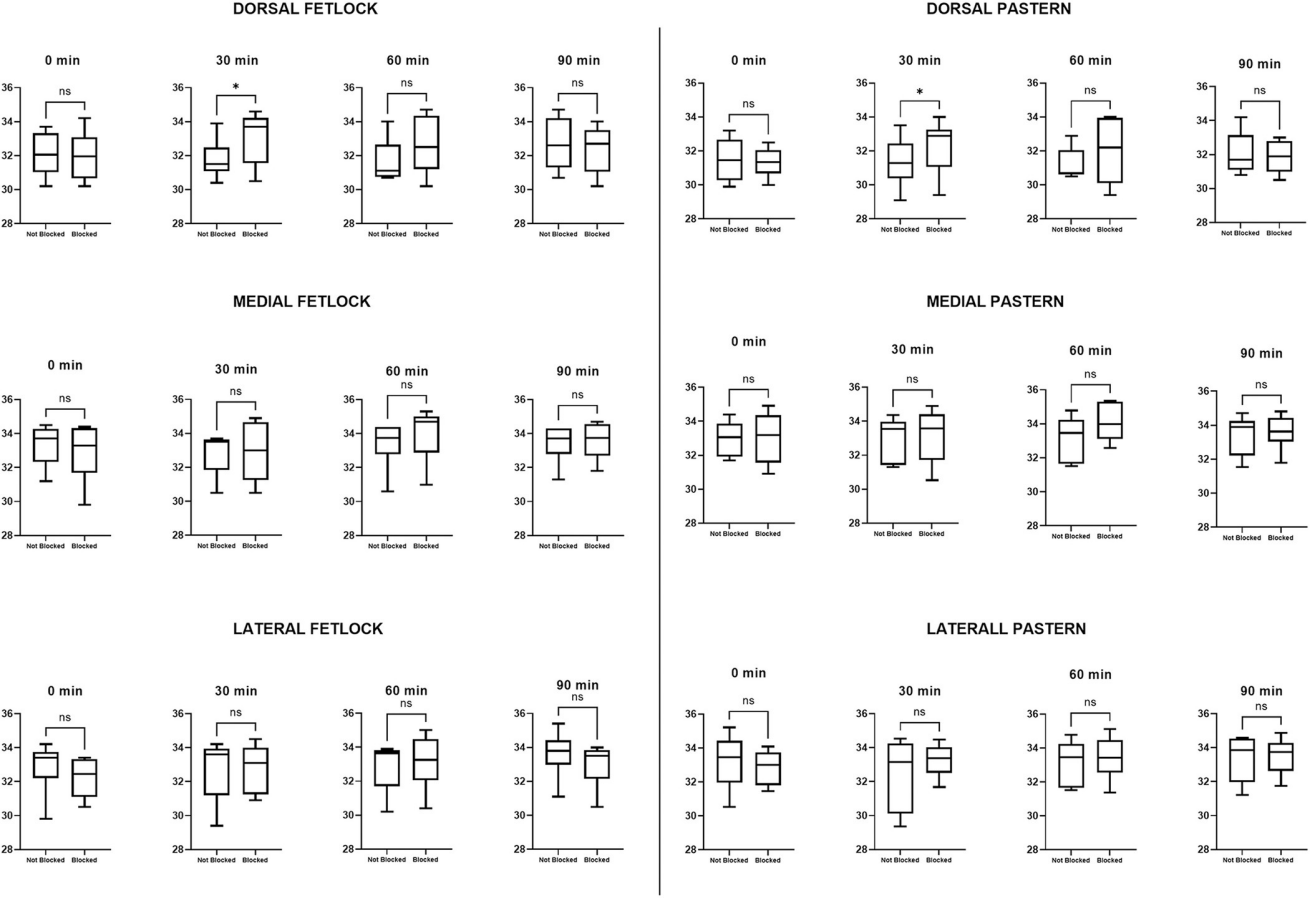
FLIR P640 maximum skin temperature between limbs. Box and whisker plots showing the maximum skin temperature in each limb for the areas of the dermatome of the median nerve taken by the P640 camera. The line indicates the median, the box the interquartile range of 25–75% and the whiskers the minimum and maximum. (ns = no significance, * = significance p<0.05).

Comparison of thermographic images between the treated and untreated limb at each ROI at different time points indicated no significant difference between skin temperatures using the FLIR One® (Table 3). The P640 analysis did result in a statistical difference in median skin temperature between treated and untreated limb for the dorsal pastern and dorsal fetlock at 30 minutes (Table 4). Box and whisker plots for differences in skin temperature between blocked and not blocked limbs are shown in Figs 5 and 6.

**Table 3:** Median and range of maximum skin temperature for different regions of the dermatome of the median nerve for the FLIR One® camera compared over time between the untreated limb (Not Blocked) and the treated limb (Blocked).

ROI	Time (minutes)	Not Blocked (°C)	Blocked (°C)	P value
Dorsal Fetlock	0	30.60 (26.60–32.30)	30.20 (25.90–32.60)	0.1875
	30	31.20 (29.70–32.20)	32.55 (25.90–33.90)	0.4375
	60	32.20 (31.20–35.10)	34.50 (30.70–35.80)	0.0938
	90	32.00 (31.00–35.20)	33.45 (30.50–35.80)	0.1875
Medial Fetlock	0	34.20 (31.90–35.40)	34.00 (30.30–35.70)	0.7188
	30	34.30 (32.90–35.00)	34.05 (30.30–35.60)	0.6250
	60	34.60 (31.90–35.45)	35.75 (33.50–36.70)	0.3125
	90	34.30 (33.00–36.00)	34.90 (32.50–36.00)	0.5000
Lateral Fetlock	0	34.45 (30.50–35.50)	33.80 (31.40–34.20)	0.3125
	30	34.30 (30.70–34.90)	34.05 (30.30–35.60)	0.9062
	60	34.45 (32.90–35.60)	33.75 (32.00–35.00)	0.0625
	90	33.55 (31.10–35.50)	34.65 (32.60–35.90)	0.3125
Dorsal Pastern	0	30.95 (26.90–32.60)	31.15 (25.90–32.50)	0.2188
	30	31.80 (30.30–32.40)	31.60 (25.90–33.10)	>0.9999
	60	32.70 (31.00–35.30)	33.50 (30.40–35.10)	>0.9999
	90	32.55 (30.80–35.80)	33.00 (30.50–35.20)	0.6875
Medial Pastern	0	33.11 (31.00–35.10)	33.59 (31.00–35.10)	0.6875
	30	33.30 (31.74–34.50)	34.00 (32.00–35.25)	0.4688
	60	33.86 (30.61–34.90)	35.65 (32.50–36.40)	0.1875
	90	33.66 (32.20–35.30)	34.47 (32.20–36.00)	0.4688
Lateral Pastern	0	32.75 (30.80–36.10)	33.80 (30.80–34.33)	>0.9999
	30	33.50 (30.80–34.62)	33.97 (30.70–35.10)	0.6875
	60	34.00 (32.30–35.90)	33.70 (32.30–35.75)	0.5000
	90	30.60 (31.70–36.20)	34.57 (31.70–36.40)	0.4375

The p value is a result of the Wilcoxon Test indicating a difference between the median skin temperatures of each limb (* = significance p<0.05).

**Table 4:** Median and range of maximum skin temperature for different regions of the dermatome of the median nerve for the FLIR P640 camera compared over time between the untreated limb (Not Blocked) and treated limb (Blocked).

ROI	Time (minutes)	Not Blocked (°C)	Blocked (°C)	P value
Dorsal Fetlock	0	32.05 (30.20–33.70)	31.95 (30.20–34.20)	0.6250
	30	31.50 (30.40–33.90)	33.70 (30.50–34.60)	**0.0312** *
	60	31.20 (30.70–34.00)	33.03 (30.20–34.70)	0.1250
	90	32.25 (30.70–34.70)	32.85 (30.20–34.00)	0.2500
Medial Fetlock	0	33.70 (31.20–34.50)	33.30 (29.80–34.40)	0.4375
	30	33.55 (30.50–34.45)	33.55 (30.50–34.90)	0.7500
	60	33.75 (30.60–34.40)	34.70 (31.00–35.30)	0.2188
	90	33.70 (31.30–34.30)	33.75 (31.80–34.70)	0.5000
Lateral Fetlock	0	33.40 (29.80–34.20)	32.45 (30.50–33.40)	0.2500
	30	33.30 (29.40–33.70)	33.30 (30.90–34.50)	0.8750
	60	33.65 (30.20–33.90)	33.25 (30.40–34.00)	>0.9999
	90	33.80 (31.10–34.10)	33.50 (30.50–34.00)	0.0625
Dorsal Pastern	0	31.45 (29.90–33.20)	31.35 (30.00–32.50)	0.5625
	30	31.30 (29.10–33.50)	32.90 (29.40–34.00)	**0.0312** *
	60	30.95 (30.50–32.90)	32.33 (29.40–34.00)	0.4375
	90	31.55 (30.80–34.20)	31.70 (30.50–33.00)	0.6875
Medial Pastern	0	33.06 (31.70–34.39)	33.18 (30.91–34.91)	0.8438
	30	33.57 (31.30–34.52)	33.23 (30.53–34.90)	0.8750
	60	33.89 (31.07–34.64)	33.97 (32.57–35.34)	0.1562
	90	33.89 (31.54–34.71)	33.63 (31.79–34.81)	>0.9999
Lateral Pastern	0	33.47 (30.53–35.21)	33.01 (31.46–34.09)	0.3125
	30	33.12 (29.35–34.52)	33.50 (31.68–34.61)	0.3750
	60	33.46 (31.52–34.78)	33.42 (31.38–35.12)	>0.9999
	90	33.85 (31.22–34.59)	33.74 (31.74–34.86)	0.8438

The p value is a result of the Wilcoxon Test indicating a difference between the median skin temperatures of each limb (* = significance p<0.05).

**Fig 7:**
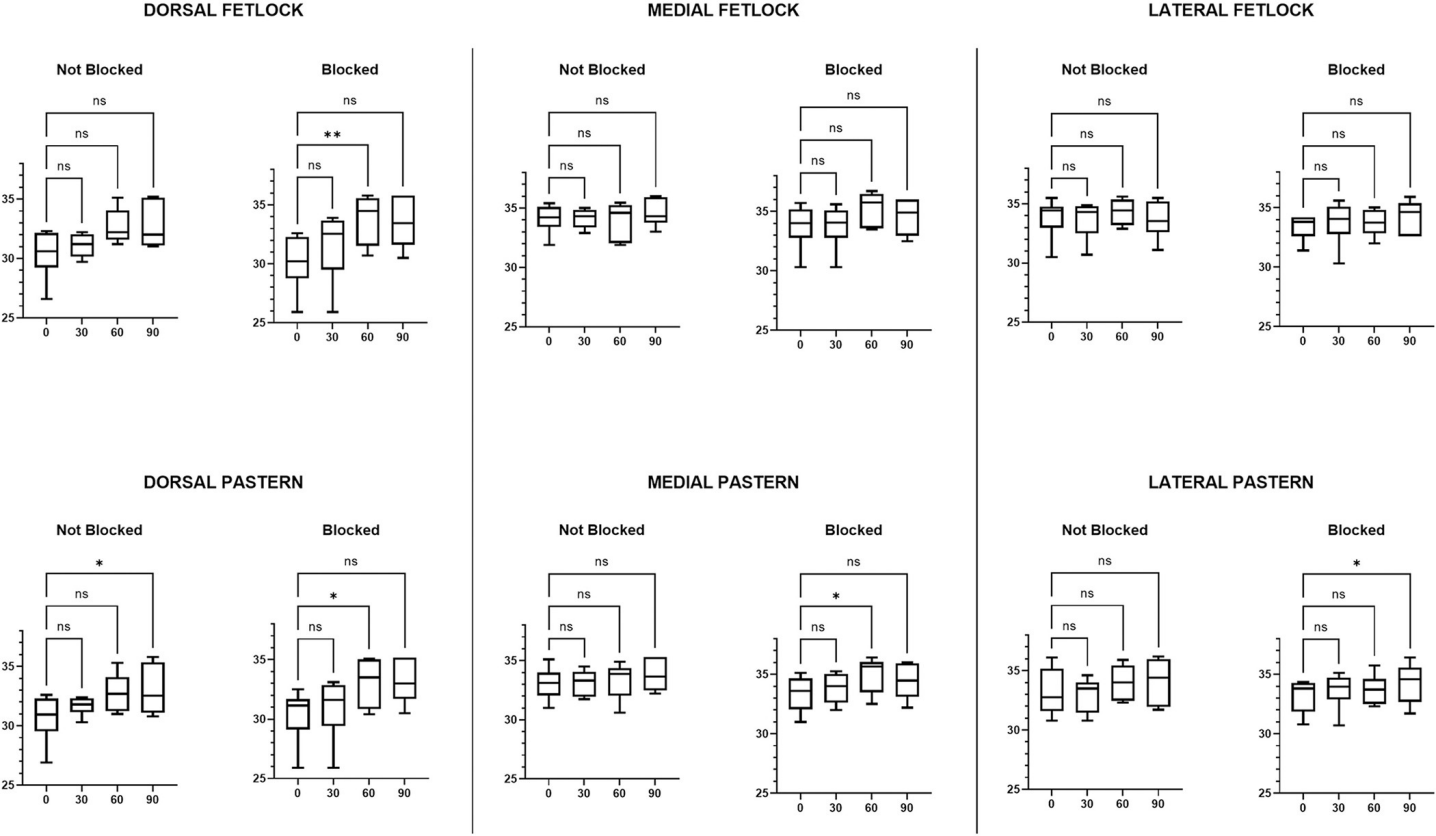
FLIR One® maximum skin temperature over time. Box and whisker plots showing the comparison in maximum skin temperature over time between each limb for the areas of the dermatome of the median nerve taken by the FLIR One® camera. The line indicates the median, the box the interquartile range of 25–75% and the whiskers the minimum and maximum. (ns = no significance, * = significance p<0.05).

**Fig 8:**
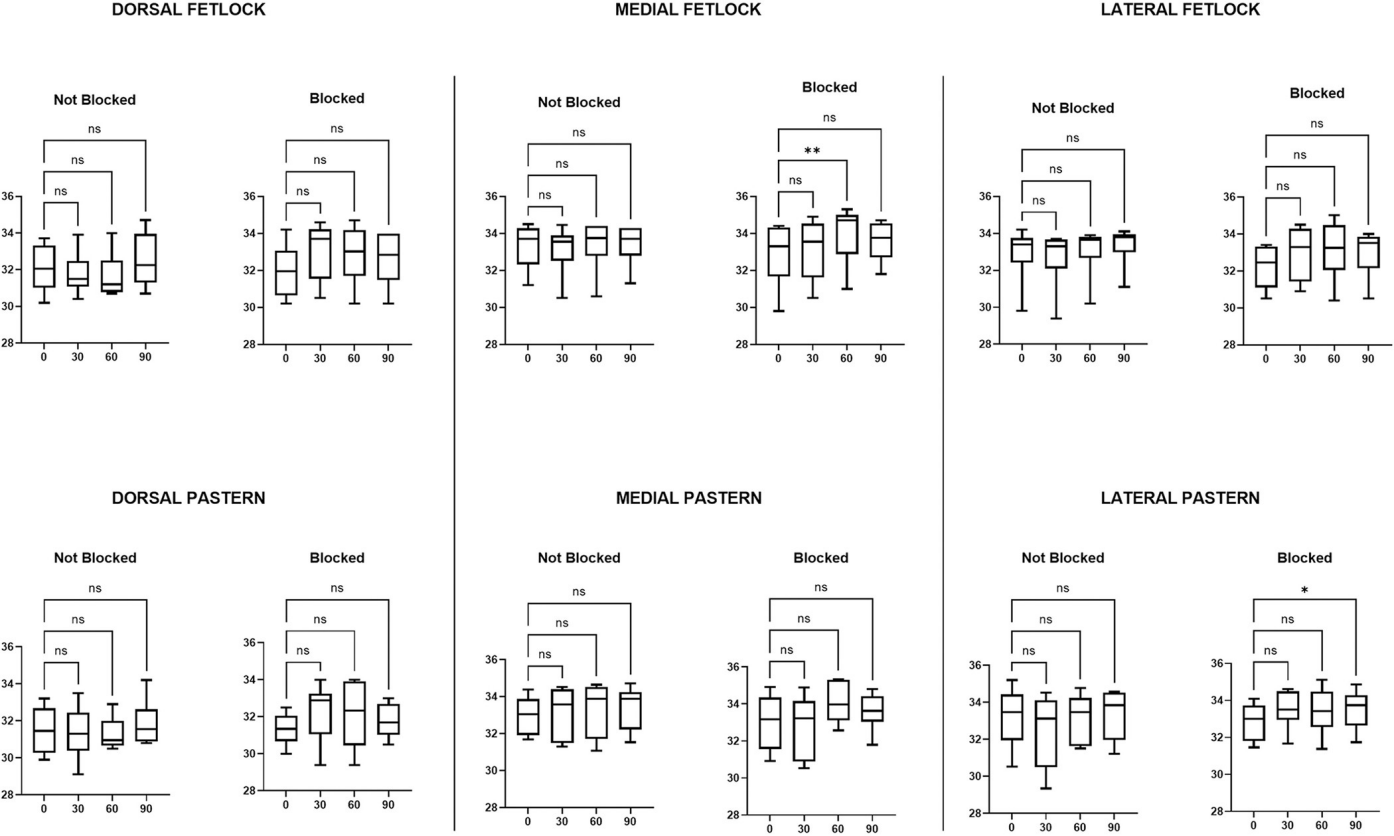
Flir P640 maximum skin temperature over time. Box and whisker plots showing the comparison in maximum skin temperature over time between each limb for the areas of the dermatome of the median nerve taken by the P640 camera. The line indicates the median, the box the interquartile range of 25–75% and the whiskers the minimum and maximum. (ns = no significance, * = significance p<0.05).

The FLIR One® Friedman analysis demonstrated a significant difference in skin surface temperature of the treated limb (blocked) at 0 versus 60 minutes with an increase in temperature at 60 minutes post nerve block in the dorsal fetlock, dorsal, medial and lateral pastern regions, as well as at 90 minutes post nerve block in the lateral pastern area (Table 5 and Fig 7). Imaging of the unblocked limb with the FLIR One® camera also had a significant increase in skin surface temperature in the dorsal pastern when comparing 0 versus 90 minutes (Table 5 and Fig 7). Comparison of skin surface temperature of images obtained with the P640 camera showed a statistically significant increase in skin temperature in the medial region of the fetlock at 60 minutes and in the lateral pastern at 90 minutes post median nerve block (Table 6 and Fig 8). In contrast to results obtained from the FLIR One® camera, no significant differences in skin surface temperature were observed with the images obtained with the P640 camera for the limb without perineural anaesthesia of the median nerve (Table 6 and Fig 8).

**Table 5:** Comparisons of the medians of maximum temperature (°C) for the baseline (0 minutes) and after (30, 60 and 90 minutes) for the FLIR One® camera in each limb (blocked and not blocked).

	BLOCKED				NOT BLOCKED			
**ROI**	**Median (IQR)** **(Baseline T = 0)**		**Median (IQR)**	**P value**	**Median (IQR) (Baseline T = 0)**		**Median (IQR)**	**P value**
Dorsal Fetlock		vs 30	32.55 (25.90–33.90)	0.6563		vs 30	31.20 (29.70–32.20)	>0.9999
	30.20 (25.90–32.60)	vs 60	34.50 (30.70–35.80)	0.0052**	30.60 (26.60–32.30)	vs 60	32.20 (31.20–35.10)	0.0760
		vs 90	33.45 (30.50–35.80)	0.0566		vs 90	32.00 (31.00–35.20)	0.0760
Medial Fetlock		vs 30	34.05 (30.30–35.60)	>0.9999		vs 30	34.30 (32.90–35.00)	>0.9999
	34.00 (30.30–35.70)	vs 60	35.75 (33.50–36.70)	0.0760	34.20 (31.90–35.40)	vs 60	34.60 (31.90–35.45)	>0.9999
		vs 90	34.90 (32.50–36.00)	>0.9999		vs 90	34.30 (33.00–36.00)	0.9429
Lateral Fetlock		vs 30	34.05 (30.30–35.60)	0.6563		vs 30	34.30 (30.70–34.90)	>0.9999
	33.80 (31.40–34.20)	vs 60	33.75 (32.00–35.00)	>0.9999	34.45 (30.50–35.50)	vs 60	34.45 (32.90–35.60)	>0.9999
		vs 90	34.65 (32.60–35.90)	0.6563		vs 90	33.55 (31.10–35.50)	0.9429
Dorsal Pastern		vs 30	31.60 (25.90–33.10)	>0.9999		vs 30	31.80 (30.30–32.40)	>0.9999
	31.15 (25.90–32.50)	vs 60	33.50 (30.40–35.10)	**0.0156** *	30.95 (26.90–32.60)	vs 60	32.70 (31.00–35.30)	0.0760
		vs 90	33.00 (30.50–35.20)	0.0566		vs 90	32.55 (30.80–35.80)	**0.0417** *
Medial Pastern		vs 30	34.00 (32.00–35.25)	0.7907		vs 30	33.30 (31.74–34.50)	>0.9999
	33.59 (31.00–35.10)	vs 60	35.65 (32.50–36.40)	**0.0100** *	33.11 (31.00–35.10)	vs 60	33.86 (30.61–34.90)	>0.9999
		vs 90	34.47 (32.20–36.00)	0.0760		vs 90	33.66 (32.20–35.30)	0.0760
Lateral Pastern		vs 30	33.97 (30.70–35.10)	0.7907		vs 30	33.50 (30.80–34.62)	>0.9999
	33.80 (30.80–34.33)	vs 60	33.70 (32.30–35.75)	0.5391	32.75 (30.80–36.10)	vs 60	34.00 (32.30–35.90)	0.2806
		vs 90	34.57 (31.70–36.40)	**0.0417** *		vs 90	30.60 (31.70–36.20)	0.9429

P value is based on the Friedman analysis. (* = significance p<0.05).

**Table 6:** Comparisons of the medians of maximum temperature (°C) for the baseline (0 minutes) and after (30, 60 and 90 minutes) for the P640 camera in each limb (blocked and not blocked).

	BLOCKED				NOT BLOCKED			
**ROI**	**Median (IQR) (Baseline T = 0)**		**Median (IQR)**	**P value**	**Median (IQR) (Baseline T = 0)**		**Median (IQR)**	**P value**
Dorsal Fetlock		vs 30	33.70 (30.50–34.60)	0.1009		vs 30	31.50 (30.40–33.90)	>0.9999
	31.95 (30.20–34.20)	vs 60	33.03 (30.20–34.70)	0.3526	32.05 (30.20–33.70)	vs 60	31.20 (30.70–34.00)	>0.9999
		vs 90	32.85 (30.20–34.00)	>0.9999		vs 90	32.25 (30.70–34.70)	>0.9999
Medial Fetlock		vs 30	33.55 (30.50–34.90)	0.7907		vs 30	33.55 (30.50–34.45)	0.7907
	33.30 (29.80–34.40)	vs 60	34.70 (31.00–35.30)	0.0076**	33.70 (31.20–34.50)	vs 60	33.75 (30.60–34.40)	>0.9999
		vs 90	33.75 (31.80–34.70)	0.1009		vs 90	33.70 (31.30–34.30)	>0.9999
Lateral Fetlock		vs 30	33.30 (30.90–34.50)	0.1720		vs 30	33.30 (29.40–33.70)	>0.9999
	32.45 (30.50–33.40)	vs 60	33.25 (30.40–34.00)	0.5391	33.40 (29.80–34.20)	vs 60	33.65 (30.20–33.90)	0.4383
		vs 90	33.50 (30.50–34.00)	0.2806		vs 90	33.80 (31.10–34.10)	0.3526
Dorsal Pastern		vs 30	32.90 (29.40–34.00)	0.1009		vs 30	31.30 (29.10–33.50)	>0.9999
	31.35 (30.00–32.50)	vs 60	32.33 (29.40–34.00)	>0.9999	31.45 (29.80–34.20)	vs 60	30.95 (30.50–32.90)	0.7907
		vs 90	31.70 (30.50–33.00)	>0.9999		vs 90	31.55 (30.80–34.20)	>0.9999
Medial Pastern		vs 30	33.23 (30.53–34.90)	>0.9999		vs 30	33.57 (31.30–34.52)	>0.9999
	33.18 (30.91–34.91)	vs 60	33.97 (32.57–35.34)	0.1325	33.06 (31.70–34.39)	vs 60	33.89 (31.07–34.64)	0.5391
		vs 90	33.63 (31.79–34.81)	>0.9999		vs 90	33.89 (31.54–34.71)	0.7907
Lateral Pastern		vs 30	33.50 (31.68–34.61)	0.1325		vs 30	33.12 (29.35–34.52)	0.1009
	33.01 (31.46–34.09)	vs 60	33.42 (31.38–35.12)	0.2209	33.47 (30.53–35.21)	vs 60	33.46 (31.52–34.78)	>0.9999
		vs 90	33.74 (31.74–34.86)	**0.0417** *		vs 90	33.85 (31.22–34.59)	>0.9999

P value is based on the Friedman analysis. (* = significance p<0.05).

### 3.3. Correlation between FLIR One® and P640

**Fig 9:**
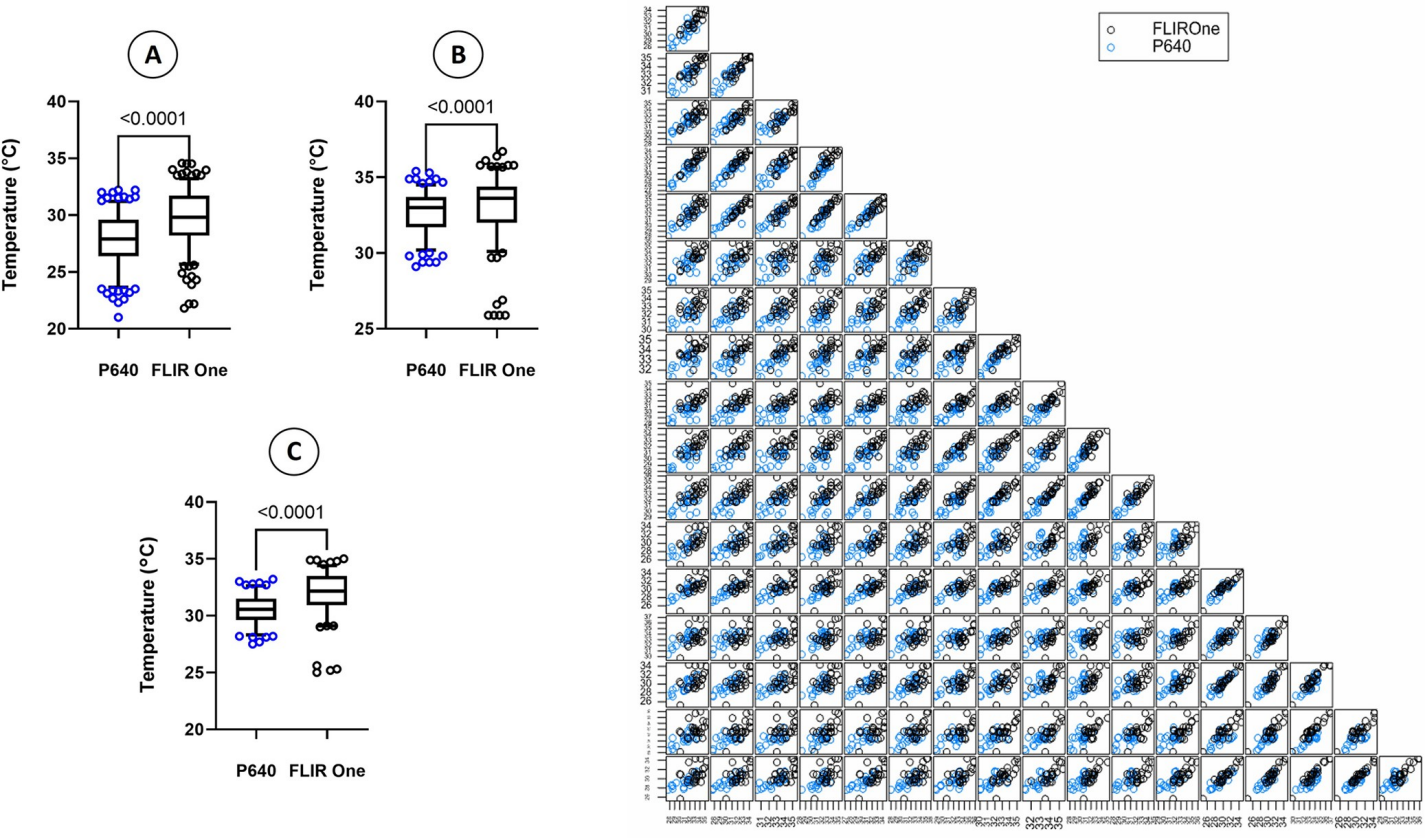
Skin surface temperature between FLIR P640 and FLIR One® infrared thermography cameras. Wilcoxon matched-pairs exact signed rank test (Left) of skin surface temperature values (Mean, 95% CI) obtained with both cameras for minimum (A), maximum (B) and average (C) temperature points. Scatterplot of scores by the two cameras across 0–60 minutes of observations (Right). Skin surface temperature is overall higher for images obtained with the FLIR One® camera compared to those obtained using the P640 camera.

The Spearman’s r correlation between the two cameras indicates a strong agreement for the minimum, maximum and average temperatures in the ROI measured in this study (Table 7). When looking at the temperature values between the two cameras, FLIR One® has a wider temperature range and temperatures trended higher than skin temperatures recorded using the P640 camera (Fig 9).

**Table 7:** Spearman’s r correlation for minimum, maximum and average temperature points across 0–60 minutes of observations between the FLIR One® and P640 camera.

Temperature (°C)	Spearman r	95% CI	P value
Minimum	0.7508	0.6869–0.8032	<0.0001*
Maximum	0.7054	0.6254–0.7707	<0.0001*
Average	0.7901	0.7160–0.8466	<0.0001 *

Spearman’s r: 1 indicating perfect agreement, 0.7–0.9 strong, 0.4–0.6 moderate, 0.1–0.3 weak and 0 indicating zero agreement [10].

## 4. Discussion

Infrared thermography provides an accurate, quantifiable, non-invasive measure and map of skin surface temperature. Skin surface temperatures are variable and change according to cutaneous blood flow and this was the basis of our hypothesis that thermography can be used to determine if a high regional nerve block was accurately performed since cutaneous blood flow should increase within the nerve’s dermatome for sensation in response to anaesthesia of a peripheral nerve. Results of the current study indicate that IR thermography can be used to detect the presence of a median nerve block based on skin surface temperature within the nerve’s dermatome. Results of this study are in agreement with a previous study that demonstrated, using thermography, an increase in skin surface temperature after performing median, ulnar, peroneal and tibial nerve blocks [3].

When performing diagnostic nerve blocks, anaesthesia of peripheral nerves is usually initiated in the distal portion of the limb and proceeds proximally if gait is not significantly improved after a nerve block. The current study demonstrated a subjective and objective increase in skin surface temperature after a high regional nerve block (median nerve), but the impact that a distal limb nerve blocks might have on skin temperature prior to performing a high regional nerve block was not investigated. In an equine study that examined the effects of a distal limb (abaxial sesamoid) nerve block on thermographic patterns, there were no changes in skin temperature [11]. The influence that distal limb nerve blocks would have on thermographic patterns of a high regional nerve block, however, remains to be investigated. Figueiredo, Dzyekanski [12] found that intrasynovial injections of bupivacaine in horses caused increases in skin temperature that were noticeable via thermography which further supports the hypothesis of loss or decrease of sympathetic function with subsequent vasodilation after perineural injection of local anaesthetic. A study using thermography to evaluate skin temperature of dogs after epidural anesthesia and femoral-sciatic nerve blocks contradicts this hypothesis [13]. The researchers found no changes in skin temperature in the paws or limbs of the dogs after they received successful regional nerve blocks (based on skin sensitivity within the nerve’s dermatome). In contrast, IR thermography was deemed useful for detection of a successful sciatic nerve block in people by demonstrating a significant increase in skin temperature within the nerve’s dermatome [4]. Inconsistencies in the resulting thermographic patterns after regional nerve blocks could be a result of species variation as well as differences between the methods used to acquire images and the software used for temperature analysis.

In the current study, when comparing baseline skin temperature to those after a median nerve block, assessors could subjectively determine the presence of a nerve block from images obtained with the FLIR P640 camera. It was possible to determine an increase in skin temperature in the dermatome of the median nerve as well as determine which limb received the nerve block. A previous study that subjectively evaluated images similar to those in our study was also successful in detecting increases in skin temperature on an IR thermogram [6]. The evaluators in our study had different levels of experience in thermography. One of the evaluators (GFA) had previous experience with IR thermography which was reflected by a higher rate of success of detecting an elevated cutaneous temperature in the dermatome of the median nerve. In contrast, one of the evaluators had no previous experience evaluating thermographic images and had less success in detecting the presence of a median nerve block. Therefore, it is reasonable to conclude that specific training in thermography evaluation and knowledge of nerve dermatomes is important and can influence interpretation and success rate in detecting the accuracy of a nerve block using IR thermography. Subjective evaluation of images obtained with the FLIR One® camera did not undergo statistical analysis due to the lack of thermal detail. The FLIR One® camera in our study yielded significantly lower quality images when compared to the FLIR P640 which was likely due to its fixed focus and limited thermal sensitivity as found in a study comparing equine thermograms produced by five different cameras [14]. It is therefore unknown if assessors would be able to correctly determine the presence of a median nerve block using the FLIR One® camera.

The data obtained using the P640 camera for objective assessment, showed significant increases in skin surface temperature from baseline in the medial aspect of the fetlock and lateral pastern after the median nerve block. Using the FLIR One® camera, significant elevation in skin temperature from baseline was noted in the dorsal fetlock and dorsal, lateral and medial aspects of the pastern. Although images obtained with both cameras showed objective increases in skin temperatures in the blocked limb, the FLIR One® also detected an increase in skin temperature in the non-treated leg which is likely due to the poor output stability and optics of this device when compared to higher resolution infrared cameras [14]. Our results found that thermographic images and data obtained with a smartphone camera (FLIR One®) and the high-end camera (FLIR P640) had strong rank correlation. However, the FLIR One® had a wider temperature range and trended higher when compared to the high-end IR camera. This finding agrees with a previous study which concluded that the FLIR One® camera produced warmer thermograms when compared to four other devices [14]. Agreement between the FLIR One® and a high-end IR FLIR camera have been previously demonstrated as seen in foot temperatures of diabetic patients, with both cameras demonstrating excellent agreement and with the smartphone camera being as accurate as the high-end camera for the detection of changes in cutaneous temperature [15]. It is noteworthy that the study aforementioned did not established if the devices were appropriately calibrated which could have interfered with the result output [14]. Despite showing an agreement with a high-end resolution infrared device, the FLIR One® camera didn’t differentiate the limbs with or without perineural anaesthesia of the median nerve in the current investigation. Based on the results herein, care needs to be taken when using the FLIR One® for exact cutaneous temperature measurement in horses. Rather, a trend of cutaneous temperature overtime might be more accurately interpreted.

Several limitations arise from the current study including the small sample size in addition to a non-normal distribution of the data. The data set for subjective analysis was less than 20 images, which weakens the assumptions on the distribution of kappa statistic. Therefore, a one-sample test for proportion was used. Furthermore, lameness resolution as a result of a successful median nerve block was not investigated and could have strengthened the study by further verifying the accuracy of the median nerve block. Although it was assumed that the median nerve blocks were accurately performed in our study, a successful nerve block was determined based on loss of reaction to noxious skin stimulation in the dermatome of the median nerve along with a corresponding increase in skin temperature in the same region. It is possible that deeper structures were not anaesthetised, and further investigation should involve a correlation of a high regional nerve block with resolution of lameness along with increased skin temperature in the region of the nerve’s dermatome. Tissue sensation is supplied by different afferent fibres within the nerve, each of which may require a different amount of time to become fully anaesthetized by the local anaesthetic. A typical progression of anaesthesia after a regional nerve block is the loss of sympathetic function, followed by a decrease or loss of sensation to touch and temperature, skin prick and deep pressure sensation, and finally loss of motor function [16]. Differential blockade can result in loss of deep pain without a loss of touch [2]. For the same reason, there may be a time-lapse between removal of skin sensation and blockade of sensitivity to deep pressure (e.g., sensitivity to application of hoof testers or sensitivity to a flexion test) in response to regional anaesthesia. Therefore, we could not be certain that after administering regional anaesthesia, deeper structures were desensitized even though skin sensation has been abolished demonstrated by lack of response to a skin pinch test. Another major limitation of this study was the manual annotation for ROI which might allow for operator error and lack of consistency of ROI selection between different images and between cameras [14]. Additionally, selection of ROI on the FLIR Tools program did not allow for margins to be traced around median nerve dermatome. Therefore, areas for average, minimum and maximum temperature were selected using either the circle or rectangle area selection tools which may have inadvertently included points outside of the nerve dermatome. For this reason, the maximum temperature was used for objective analysis as to avoid the possibility of the data being skewed if including areas with lower temperatures such as the background of the image. The differences in specifications between the two cameras such as detector array size and sensitivity, lens size and focal length may have also contributed to variability in the field of view, size of source effect as well as inconsistency in pixels in the anatomical ROI between cameras [17]. Although all images were acquired in a temperature-controlled room, the lowest constant temperature that the room allowed for was 20 degrees Celsius which is 2 degrees higher than the recommended temperature of less than 18 degrees Celsius when evaluating vasodilator agents [18]. It is possible that a natural vasodilation of the limbs occurred at 20 degrees Celsius which could lead to a lower effect of the perineural anaesthesia in the thermographic images. Lastly, horses with different hair coats were used and the effect of hair coat on skin surface temperature and the optimal coat emissivity was not investigated in the present study and the information is not currently available in the literature.

## 5. Conclusion

Infrared thermography can be a useful, non-invasive method for detecting perineural anaesthesia of the median nerve in healthy horses. The findings indicate subjective and objective analysis of images obtained with the FLIR P640 can detect temperature increases in the median nerve dermatome after perineural anaesthesia. Although comparisons of skin temperature obtained with the high-end resolution IR camera and the smartphone IR device showed good correlation between the two cameras, care needs to be taken when using the smartphone device in horses as it reports overall higher skin temperature. Furthermore, the FLIR One® device detected an increase in skin surface temperature in both treated and non-treated legs and should not be used for assessment of a median nerve block. Further investigation of objective and subjective analysis of IR thermography imaging after perineural anaesthesia of other high regional nerve blocks is warranted.

## Supporting information

S1 File FLIR thermography data. (XLSX)
